# Mechanistic modelling supports entwined rather than exclusively competitive DNA double-strand break repair pathway

**DOI:** 10.1038/s41598-019-42901-8

**Published:** 2019-04-23

**Authors:** S. P. Ingram, J. W. Warmenhoven, N. T. Henthorn, E. A. K. Smith, A. L. Chadwick, N. G. Burnet, R. I. Mackay, N. F. Kirkby, K. J. Kirkby, M. J. Merchant

**Affiliations:** 1 Division of Cancer Sciences, Faculty of Biology, Medicine and Health, The University of Manchester, Manchester, UK; 2 Christie Medical Physics and Engineering, The Christie NHS Foundation Trust, Manchester, UK; 3 The Christie NHS Foundation Trust, Manchester Academic Health Science Centre, Manchester, UK

**Keywords:** Computational models, Radiotherapy, Double-strand DNA breaks, Homologous recombination, Non-homologous-end joining

## Abstract

Following radiation induced DNA damage, several repair pathways are activated to help preserve genome integrity. Double Strand Breaks (DSBs), which are highly toxic, have specified repair pathways to address them. The main repair pathways used to resolve DSBs are Non-Homologous End Joining (NHEJ) and Homologous Recombination (HR). Cell cycle phase determines the availability of HR, but the repair choice between pathways in the G2 phases where both HR and NHEJ can operate is not clearly understood. This study compares several *in silico* models of repair choice to experimental data published in the literature, each model representing a different possible scenario describing how repair choice takes place. Competitive only scenarios, where initial protein recruitment determines repair choice, are unable to fit the literature data. In contrast, the scenario which uses a more entwined relationship between NHEJ and HR, incorporating protein co-localisation and RNF138-dependent removal of the Ku/DNA-PK complex, is better able to predict levels of repair similar to the experimental data. Furthermore, this study concludes that co-localisation of the Mre11-Rad50-Nbs1 (MRN) complexes, with initial NHEJ proteins must be modeled to accurately depict repair choice.

## Introduction

Radiation is known to damage DNA both directly and indirectly. This DNA damage elicits a range of biological responses to ensure the preservation of genome integrity. If left un-repaired, DNA damage can lead to cell death^1^. Therefore, a key function of the DNA Damage Response (DDR) is the attempt to preserve the cell’s function. There are several repair pathways available for DNA repair, each with its own collection of biological consequences. For example, Base Excision Repair (BER) is responsible for the removal of non-helix-distorting base lesions, whereas Nucleotide Excision Repair (NER) is responsible for removal of the bulky helix-distorting lesions. Other pathways are thought to compete for the same types of damage, such as the availability of both Non-Homologous End Joining (NHEJ) and Homologous Recombination (HR) for the repair of DNA Double Strand Breaks (DSBs) during the G2 phase of the cell cycle. There are also “back-up” pathways for situations where primary repair pathways are unavailable: for example Microhomology-Mediated End Joining (MMEJ) is sometimes used instead of NHEJ, but will often cause large deletion mutations. This variety of repair pathways, acting in unison to address DNA damage, accounts for the observed biological resilience in dealing with natural levels of endogenous and exogenous damage resulting from everyday life.

The exogenous generation of DNA damage is not intrinsically bad; it is utilised in the form of cancer treatments where the aim is to achieve cell death within a cancer target while avoiding too much damage to healthy surrounding tissue and critical organs. For instance, radiotherapy is used in the treatment of 40–50% of patients diagnosed with cancer^2,3^, because of its non-invasive ability to damage DNA and can be geographically localised. Radiotherapy works by creating high amounts of DNA damage, much greater and more complex than natural levels, leading to increased cell death. Radical radiotherapy aims to deliver enough radiation to the cancerous target volume to gain local control while minimising radiation dose to healthy tissues and critical organs and thereby avoiding compromises in quality of life. Although it is understood that elevated levels of cell death are caused through radiotherapy, there is a clinical need to understand and quantify how both DNA damage and repair impact patient treatment for different radiation modalities, energies and spectra, and dose rates.

Treatment planning is based on physical dose, with dose prescriptions guided by clinical experience. Biological effects are considered through phenomenological modelling. For example, the Linear-Quadratic model is used clinically to guide both fractionation and organ dose constraints. However, at the planning stage biological optimisation of dose deposition is not considered. While aspects of not accounting for these biological factors are counteracted by more than 100 years of experience in X-ray radiotherapy, in other treatment modalities, such as proton beam therapy, not accounting for these variations in radiobiological effectiveness (RBE) could potentially limit treatment success^4,5^. There has been a recent resurgence of interest in modelling of DNA damage and repair^6–13^, which aims to give quantifiable depictions of the DNA damage response following irradiation, with some models^7,9^ able to account for some of the aforementioned radiation properties. Some *in silico* models^10,12^ attempt to evaluate the biological response through observations of DNA damage alone, although they neglect the variations known to exist in repair function between cell type, organ type and patient^14–16^. By including models of the DNA repair, it is possible to account for some of these differences, and predict their relationship with different DNA-level endpoints (e.g. repaired, un-repaired damage and misrepair)^7^, and potentially relate these to cell fate^8^. Although more onerous, including DNA repair in *in silico* models is the first step towards achieving patient-specific, biologically optimised, treatment plans.

In radiotherapy, radiobiological models of DNA repair have typically been focused on pathways that interact with DSBs, since DSBs have a significant role in cell fate^1^. It has been established that, for a population of cells, NHEJ is the most frequently used repair pathway as it is present through all phases of the cell cycle^17,18^, whereas HR becomes more pronounced during the S and G2 phases due to the availability of a sister chromatid supplying proximal homology. Therefore, in most work, NHEJ is the focus of the modelling efforts^6–8,19^, with HR being partially or entirely omitted from models. Although HR makes up a smaller proportion of repair, several publications have suggested that HR may be preferential for the repair of proton-induced DSBs, and that in cohorts of HR impaired patients there are significant clinically observable effects^20–23^. Futhermore, HR is of interest for drug radiosensitizers, HR-deficiencies have been shown to be especially lethal when paired with a poly(ADP-ribose) polymerase (PARP) inhibitor^24,25^, which may be exploited for treatment of some cancer types. Finally, incorporation of more than one repair pathway is important as it is believed that many subsets of cancers include perturbations to various DNA repair pathways, altering their response to radiation^23^.

It is well established that the availability of prominent HR repair is regulated by the cell cycle and sister chromatid availability, which the pathway uses as a homologous template^26^. However, the processes of repair choice are less well established (i.e. during G2 where both NHEJ and HR are available). To model the mechanism of repair choice in an *in silico* step-by-step model, like the one proposed within this study, requires the explicit inclusion of the stage or stages within the repair pathway where repair choice is possible. It has been generally believed that NHEJ and HR act in a predominately competitive fashion^27^, with initial protein attachment directing repair fate. However, other repair choice models have been suggested within the literature^27–30^ and it is under these different scenarios that our *in silico* models will be compared against experimental literature data. Four scenarios are tested: Scenario A) ‘NHEJ first’ approach, Scenario B) ‘no way back’ approach, Scenario C) ‘continuous competition’ approach, and Scenario D) ‘entwined pathway’ approach. The repair pathways NHEJ and HR have different biological consequences with NHEJ being more error prone than HR^29^. Therefore, understanding how to model the repair choice *in silico* is required in any mechanistic attempt to quantify the amount and fidelity of repair which takes place for a given DNA damage pattern. It has been shown that while NHEJ and HR share some of the DSB caseload, when each pathway is removed in turn, the remaining pathway can compensate for its deficient counterpart to a differing extent^31^. It is the purpose of this paper to evaluate this complex behaviour which suggests a level of overlap between the repair pathways.

## Methods

**Figure 1:**
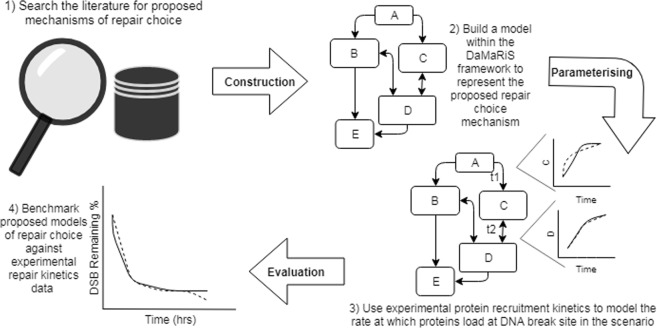
Schematic overview of this study’s methodology. (1) The literature was reviewed to establish possible mechanisms of repair choice. (2) These mechanisms were pieced together to form cohesive possible scenarios of DNA double-strand break repair choice. The proposed scenarios and the required protein recruitment steps were formed in the DaMaRiS framework. (3) The created scenarios were parameterised to allow for the addition of protein recruitment time constants which emulate protein recruitment kinetics similar to experimental literature data. (4) The produced models were used to simulate individual cells undergoing DNA repair following radiation damage and the overall repair kinetics were measured for each proposed scenario. The measured repair kinetics were then evaluated against experimental *γ*-H2AX foci data to benchmark each scenario.

The *in silico* framework used to produce the models for this study is the DNA Mechanistic Repair Simulator (DaMaRiS) framework^6,7^, developed at University of Manchester which has been built using the Monte Carlo Geant4-DNA simulation toolkit^32^ (version 10.4). The produced DaMaRiS model requires an input of the distribution of DNA DSB damage following irradiation, this was supplied by an in-house model of DNA damage^7^, but in principle could be supplied by other DNA damage models which output in the Standard DNA Damage (SDD) Data Format^33^. The DaMaRiS framework (version 0.3) is designed to be versatile, enabling the user to define, build and test models of DNA repair. The mechanistic nature of the DaMaRiS framework requires every parameter in the model should have a biological definition, thereby allowing for model alteration by the addition and/or removal of biological mechanisms as they are elucidated within the literature. The applied mechanisms, in unison, form entire repair pathways (e.g. NHEJ and HR), making DaMaRiS well suited for the multi-scenario modelling presented in this study. Each mechanistic step of the model depicts how proteins attach and react in order to process broken DNA ends from break recognition through to repair completion. The framework provides temporal and information on how the DNA damage is processed along the repair pathways at any time point. The DaMaRiS models in this study consist of NHEJ and the initial steps of HR, with the intent to add further repair pathways to provide a full depiction of the DDR at the DNA level. Since the DaMaRiS models are mechanistic, where each core step of a pathway is incorporated in the model, it is necessary to understand at which point or points the repair pathway choice is carried out (i.e. areas of repair pathway where the DNA end can transfer from being actively repaired by one available pathway to another). Furthermore, it is important to assess, in the case of repair failure, if alternative pathways become available or even enforced, as depicted in Scenario A below. This study is the first time HR has been modelled in our DNA repair work; all previous work has focused on NHEJ with no repair choice. Furthermore, we have used the model to describe protein deficient systems which are a new potential area of the DaMaRiS framework being explored. A schematic overview of the methodology process has been outlined in Fig. 1.

### Repair choice scenarios

**Figure 2:**
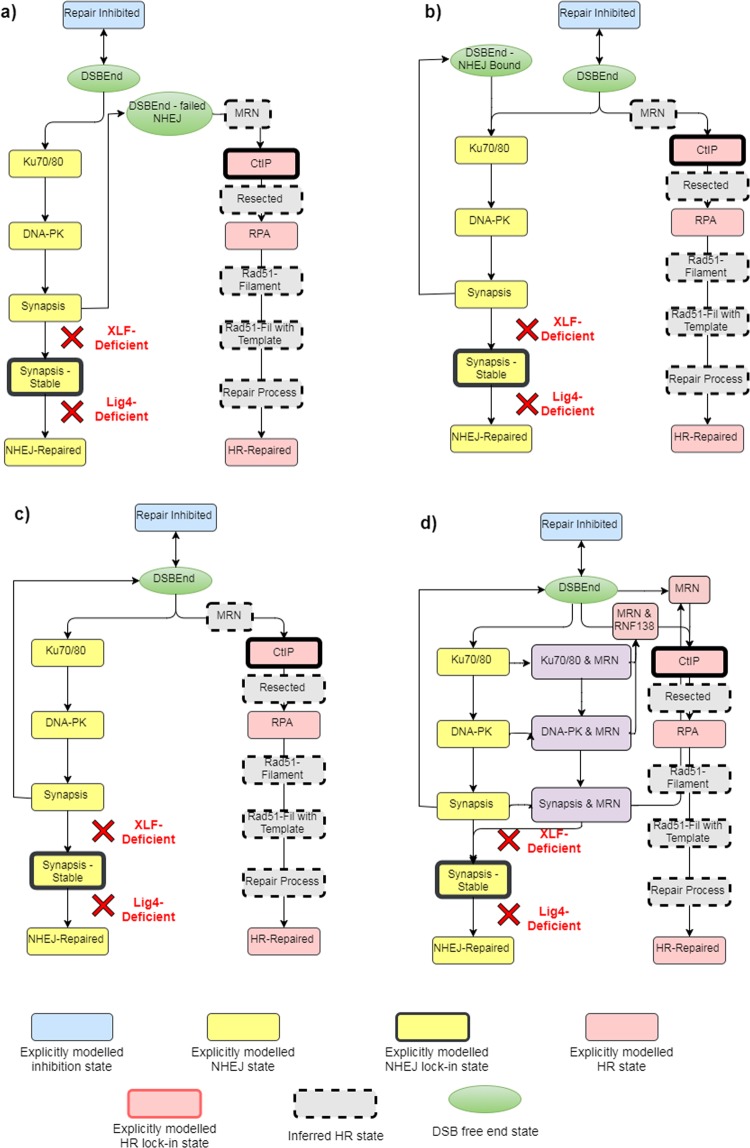
Scenario schematics – (**a**) Scenario A - NHEJ is attempted first and only upon failure can HR be attempted. (**b**) Scenario B - initial competition dictates the repair progression as there is no cross-over allowed between pathways; the pathway which has the end will be attempted until the end of simulation time. (**c**) Scenario C - initial competition dictates the attempt of repair, and upon failure, re-competition takes place for the next attempt of repair, this is repeated until the end of simulation time. (**d**) Scenario D ‘Entwined pathway approach’ - a similar competition to Scenario C with the addition of MRN co-localisation with initial NHEJ proteins, Ku dissociation and PKcs dissociation; it should be noted that dissociation of NHEJ proteins where MRN is co-localised results in the fall back to a DNA end with MRN attached. The steps in the model are either explicitly modelled (yellow NHEJ and red HR) with progression and reaction rates deduced from experimental data (Supplementary Data - Figs S1 and S2), or there are several inferred HR steps (grey with dashed external lines) which are assumed to have taken place by the time the next explicitly modelled step is reached. The emboldened steps within the schematic show points at which the DNA-end gets modified such that repair can only progress in the corresponding pathway (the point at which a DSB end is locked into the pathway). The green circular steps represent DSB free ends with none of the modelled repair proteins loaded, which occur either at the start of the simulation (i.e. as repair is about to start) or through DNA-PKcs synapsis dissociation. The blue step represents the DNA-end being inhibited from protein loading and represents aspects of end cleaning before proteins can successfully load on. Finally, the red crosses represent the progression points that are deactivated for working in a protein deficient cell system; the text of XLF- or Lig4-deficient indicates at what point the DNA end progression is removed for the corresponding system.

The repair choice scenarios (Fig. 2) modelled, as well as a brief summary of their justification, include:

A) The ‘NHEJ first’ approach - NHEJ processes so much faster than HR and is therefore always attempted first with only failure allowing HR repair to be used^28,29^.
  The concept of NHEJ being the first attempted repair pathway originates from the experimentally observed faster repair kinetics^34,35^. Therefore, this scenario is analogous to NHEJ being so much faster that it is always attempted first, rather than a competitive approach used in the other scenarios. The model was adapted so that DSBs entering the system could only progress down NHEJ, and only upon failure at the synapsis complex stage is HR attempted (i.e. DNA-PKcs auto-phosphorylation and disassociation)^35^. The HR repair following NHEJ failure was enforced (i.e. HR was the only available pathway upon NHEJ failure), removing the possibility of multiple attempts of NHEJ for the same break.
B) The ‘no way back’ approach competitive directed repair with no cross-over of pathways, the initially recruited protein locks the broken end into its corresponding repair pathway which it can either succeed or fail to repair^27^.
  NHEJ and HR repair pathways being directed by competition is a commonly used hypothesis within the literature^36,37^. However, when implementing a competition-based approach the concept of pathway cross-over (i.e. DSB ends being able to transfer from one pathway to another) needs to be addressed. In Scenario B there is no cross-over allowed; this is realised by the first protein attachment to the broken DNA end locking the end into the respective pathway, effectively inhibiting the other pathway from even attempting repair at any time point. Whilst this scenario’s description of cross-over is not explicitly stated within the literature, it does represent the commonly held assumption of repair pathway cross-over (i.e. no cross-over) shown in textbook descriptions^38^. Furthermore, the scenario acts as a point of completeness when comparing to the other cross-over description, in which, upon failure of repair, the DNA end becomes available for re-competition.
C) The ‘continious competition approach’ same as Scenario B: but upon failure of repair the end is available for competition again^30^.
  This scenario depicts NHEJ and HR competition in combination with allowed cross-over of repair pathways upon repair failure. Upon all cases of dissociation, there is re-competition between initial HR and NHEJ proteins. This re-competition enables HR proteins to have a chance to interact with isolated DNA ends which are not suitable for NHEJ but can be repaired by HR. This scenario, along with Scenario D, is seen as the least directed system and is largely dictated by protein recruitment kinetics.
D) The ‘entwined pathway’ approach: similar to Scenario C, but includes mechanisms of protein co-localisation and RNF138 mediated Ku70/80 removal.

In this scenario, additional mechanisms from the recent literature were added which resulted in a substantial change for the simulated repair kinetics and partialy addressed the issue of Ku70/80 and CtBP-interacting protein (CtIP) competition detailed above. Scenario D is similar to Scenario C as it utilises continuous competition, but includes the three additional mechanisms:

- MRN co-localisation with Ku70/80, DNA-PK complex (Ku70/80 and DNA-PKcs) and the DNA-PK synapsis complex^36,39^.
- RNF138-dependent Ku removal from the DNA ends with MRN attached to allow for an additional point of resection, thereby promoting HR^40^.
- MRN independent resection to allow for naked DSB ends to undergo resection^41^.

These additional mechanisms describe a more entwined initial DNA-repair response than the previously perceived hypothesis that exclusive competition directs the repair choice^27,30,37^.

These scenarios are assumed to span the most likely possibilities for the mechanism of repair choice. However, this field is still being actively developed; therefore the framework remains available for the evaluation of further drivers of repair choice. Aspects such as DNA density (i.e. euchromatin and heterochromatin) and damage complexity which are believed to also impact repair choice^42,43^ will be assessed independently of this study.

### Model construction

The DaMaRiS models are altered versions of one another with the removal and/or addition of available progression points from the initial DSB end, describing each of the proposed repair choice scenarios (Fig. 2). The transition from one state to another is controlled through a series of time constants acted upon by the random number generator of Geant4, generating a distribution of time delays for progression. Each time constant and its generated distribution of time delays have been derived by fitting to experimental protein recruitment kinetics, giving an accurate progression rate of step-by-step repair (see Supplementary Data - Figs S1 and S2). The only time-constant not fitted to experimental data is the progression rate between a “resected end” to “repaired by HR” (*τ*_*RR*_). Other steps, i.e. the rad51-filament formation, homology-seeking along the sister chromatid, DNA replication and double Holliday Junction resolution have not been explicitly modelled, so a combined large time constant is used instead. This loss of detail, after resection within the HR pathway, does not affect the repair choice process, since it has been established that canonical NHEJ cannot be utilised following post-resection from CtIP^44^. Although new work regarding NHEJ resection mediated repair in G1 has been proposed^45^, this study focuses in G2 where the assumption is that resection-mediated repair is predominately HR. Therefore, the time constant *τ*_*RR*_ was allowed to vary in order to achieve the best fit to the experimental data. If a scenario did not fit the experimental data whilst varying *τ*_*RR*_ it was concluded that the scenario is not fit for purpose (see Supplementary Data - Figs S3–S6).

### Model evaluation

Repair kinetics of DSBs for each scenario were compared to phosphorylated form of histone H2AX (*γ*-H2AX) foci, a commonly used experimental marker of DSBs. It is well established that on average HR takes significantly more time than NHEJ to repair a break, and this has given rise to the extensively used dual component description of slow (HR) and fast (NHEJ) repair to describe the repair rates seen^31,45^. The repair choice of each scenario will alter the ratio of HR and NHEJ and since each pathway has specific progression rates, the overall shape of the repair rate is changed. This allows the agreement between the generated data and the experimental data to be compared. Furthermore, these quantities can be altered by the scenarios whilst remaining predominately independent of protein recruitment kinetics. However, to ensure a fair comparison the recruitment kinetics have been optimised for each scenario (Supplementary Data - Figs S1 and S2), but fixed when evaluating against the experimental *γ*-H2AX foci data.

To gain further confidence for each scenario the repair rates in two deficient cell lines were also reviewed. This review was possible because the mechanistic step-by-step model can have progression points removed (inhibited) at any point along the pathway, allowing accurate depictions of different deficiencies within the same repair pathway. This results in four scenario models being produced, each with the three configurations (Fig. 2): wild-type cells - all progression points intact, XLF-deficient cells - all progression points intact excluding synapsis complex stabilisation between two DNA-PKcs-loaded ends within NHEJ^46^, and Lig4-deficient cells - all progression points intact excluding formed synapsis complex ligation creating repaired DNA through NHEJ^47^.

### Experimental benchmarks

The overall repair kinetics were compared to the *γ*-H2AX foci data extracted from Beucher *et al*.^31^ using WebPlotDigitizer (https://automeris.io/WebPlotDigitizer^48^), to evaluate the modelled repair choice scenarios. The goodness of fit was compared between the experimental data points and corresponding simulation data points. The comparison was carried out using Reduced Chi-Square (${\chi }_{red}^{2}$), Root Mean Square Error (RMSE) and Dynamic Time Warping (DTW)^49^. Both ${\chi }_{red}^{2}$ and RMSE evaluate data points at the same time point, whereas DTW can evaluate possible time discontinuities between the simulated and experimental data sets. In the Beucher *et al*. experiment, the DNA damage was induced by a 2 Gy exposure from a Cs-137 source. The Beucher *et al*. experimental data set used was from G2-phase human fibroblast (HF) and mouse embryonic fibroblasts (MEFs) cells and included variations of wild-type (WT) cells (C2886 and “WT2” for human and mouse respectively), XLF-deficient cells (2BN HF) and Lig4-deficient cells (“Lig4−/−“ MEFs). The Beucher data set was used to evaluate the simulations agreement to experiment. This is due to it being the only dataset used which has been G2 synchronised. Further experimental data was sourced and added to evaluate how the repair rate may vary through different experimental parameters. Experiments carried out by Kuhne *et al*.^50^ delivered 2 Gy of 90 kVp X-rays, filtered by 1 mm of aluminium. The data extracted from Kuhne *et al*. includes a Wild-Type Lung HF (48BR) and an additional Lig4-deficient cell line (411BR). However, the Lig4-deficient line was derived from a patient with Lig4 syndrome, which results in a prominent but not complete removal of available Lig4 protein. The experimental data extracted from Wu *et al*. 2012^51^ is for 2 Gy of 7.5 MeV protons, which corresponds to a reported linear energy transfer (LET) of 6 keV/*μ*m (calculated by the authors using SRIM), delivered to Normal Human Lung Fibroblast (NHLF) cells. All the experimental repair rates were deduced from *γ*-H2AX foci experiments and were self-normalised. The *in silico* models were configured to simulate the total amount of repair through NHEJ and HR of DSBs at 8-hours post-irradiation (matching the Beucher *et al*. data set), recording the progression of the simulation every 30 seconds. The DSB pattern used as an input for the simulation was created from the model described by Henthorn *et al*.^7^ and simulates 2 Gy to the cell nucleus using a 34 MeV mono-energetic proton beam (equivalent to a track-averaged LET of 1.77 keV/*μ*m). Relatively low LET protons were selected as experimental data has shown that the repair kinetics are similar to that of photon irradiation^52,53^.

## Results

**Figure 3:**
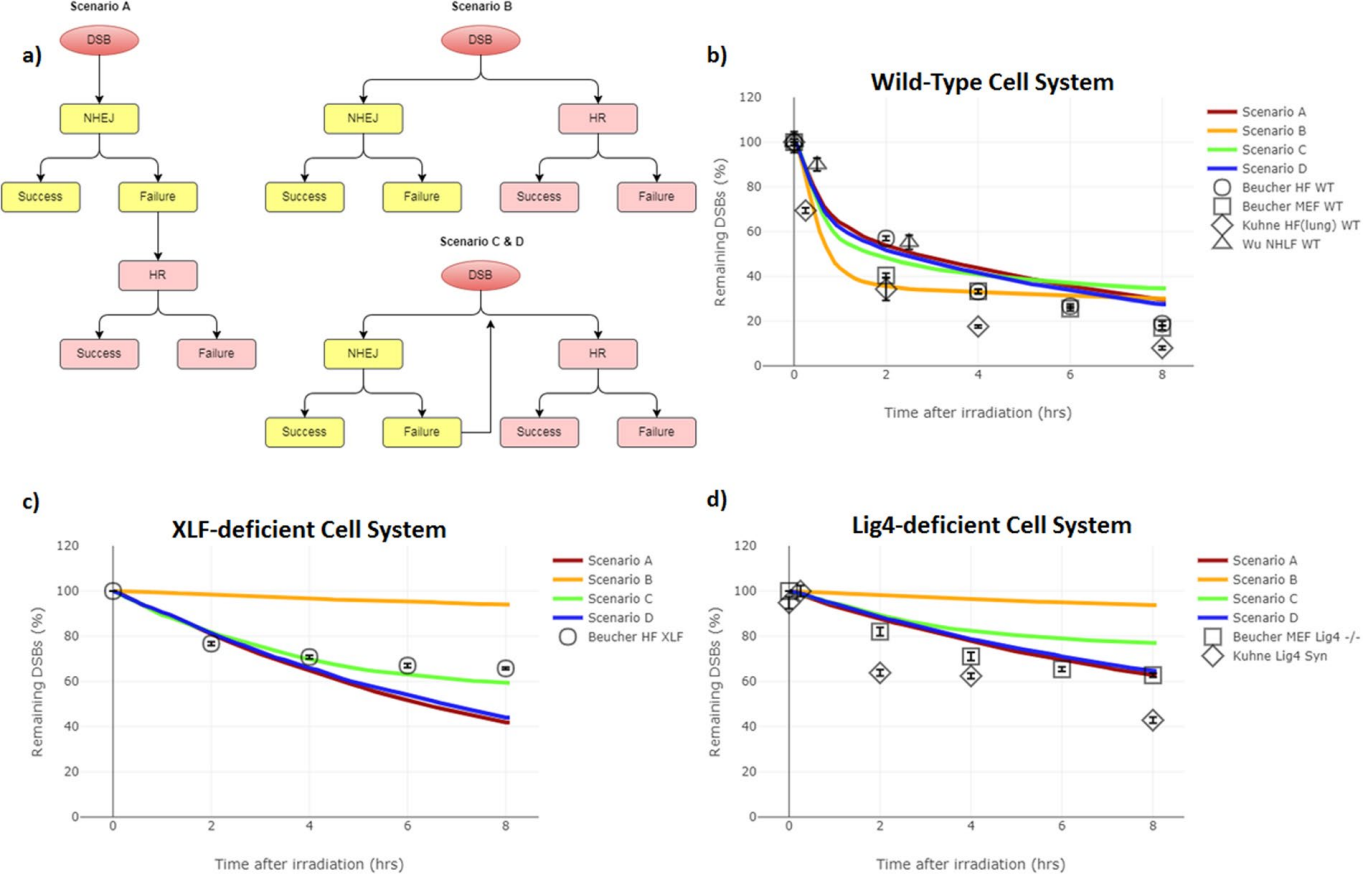
Scenario Comparison – (**a**) simplistic flow diagrams of the repair choice scenarios tested. Scenario A represents the ‘NHEJ first’ approach. Scenario B represents the ‘no way back’ approach between pathways. Scenario C represents the ‘continuous competition’ approach between pathways. Scenario D represents the ‘entwined pathway’ approach. The results for the wild-type, XLF-deficient and Lig4-deficient systems are shown in (**b**), (**c**) and (**d**) respectively. The diamond and the triangle symbols are for comparison between experimental data and are not included in the quantitative analysis of fit between simulated and experimental data. The error bars are the reported ±SEM from the experimental data set (black data points). The error in the simulated data is the ±SEM is displayed as the line width for 50 repeated simulations each with their own independent exposures on different cells.

Scenarios are analysed for best fit with the experimental data from the literature. The allowed variation in *τ*_*RR*_ was implemented to investigate if the Beucher *et al*. experimental data points at the various time points could be achieved through simulation. The ability to do so is indicative of a suitable repair choice scenario, while the deviation of fit when using an average value of *τ*_*RR*_ (Fig. 3) may be an indication of missing mechanisms within the current model (see discussion). The goodness of fit metrics for each Scenario in each cell system are presented in Table 1.

**Table 1:** Summary of goodness-of-fit metrics between simulated and the experimental Beucher data set^31^ and the purposed repair choice scenarios. Each cell system from each scenario has been evaluated against the experimental data set. For every experimental data point a corresponding simulation point was evaluated against. Reduced Chi Square (χ^2^), Root Mean Square Error (RMSE) and Dynamic Time Warping (DTW) were used to quantify the goodness-of-fit. The values presented in bold show the mean goodness-of-fit for each Scenario.

Simulated System	Reduced Chi Sq	Root Mean Square Error	Dynamic Time Warping
Scenario A - Lig4	0.42	4.31	16.16
Scenario A - XLF	4.35	13.18	49.73
Scenario A - HF WT	4.18	7.92	30.90
Scenario A - MEF WT	6.89	10.42	37.18
**Scenario A Average**	**3.96**	**8.96**	**33.49**
Scenario B - Lig4	13.61	23.38	100.59
Scenario B - XLF	13.26	23.44	102.65
Scenario B - HF WT	5.13	10.97	37.38
Scenario B - MEF WT	3.88	6.68	23.84
**Scenario B Average**	**8.97**	**16.12**	**66.11**
Scenario C - Lig4	2.82	10.63	43.37
Scenario C - XLF	0.40	4.09	16.36
Scenario C - HF WT	6.72	9.86	42.24
Scenario C - MEF WT	4.76	10.48	40.62
**Scenario C Average**	**3.68**	**8.77**	**35.65**
Scenario D - Lig4	0.61	5.16	16.19
Scenario D - XLF	3.44	11.72	44.20
Scenario D - HF WT	2.85	6.71	26.67
Scenario D - MEF WT	4.76	8.64	28.97
**Scenario D Average**	**2.92**	**8.06**	**29.01**

### Scenario A - The ‘NHEJ first’ approach

Scenario A has a relatively good agreement (${\bar{\chi }}_{red}^{2}=3.96$) with the experimental data from each tested cell system (i.e. WT, Lig4-deficient and XLF-deficient). In the WT-system (Fig. 3b) the repair rate up to 2-hours agrees with the experimental human fibroblast data and demonstrates a slower but similar shape until 8 hours. In the XLF-deficient system (Fig. 3c) the simulation predicts a higher amount of repair beyond 4 hours than is seen in the experimental data. Furthermore, the repair rate appears to be missing any bi-phasic behaviour resulting in a predominately linear result and negates the required curvature to match the experimental data. In the Lig4-deficient system (Fig. 3d) Scenario A gives the best agreement over all the tested scenarios (${\chi }_{red}^{2}=0.42$). However, this is only marginally better than Scenario D. The agreement demonstrates the requirement of Lig4 being present for final ligation and the premise of a stabilised DNA-PKcs synaptic complex^46,54^, which in the Lig4-deficient cell system would result in increased un-repaired DNA ends within the NHEJ pathway.

### Scenario B - The ‘no way back’ approach

The goodness-of-fit metrics, shown in Table 1, highlight that the concept of the ‘no way back’ approach has the least agreement with the evaluated Beucher *et al*., data set (${\bar{\chi }}_{red}^{2}=8.97$). In the WT-system (Fig. 3b) the simulated results of Scenario B demonstrates a situation where the absolute differences from the experimental points are not extensive, but the overall shape shows clear disagreement with all data sets. The simulation shows very fast repair kinetics before the 2-hour time point and a plateau beyond this. This shape is due to NHEJ dominating the system and DNA ends which become isolated, without a partner end to form a synaptic, will remain as such for the rest of the simulation. In both the XLF- and Lig4-deficient systems (Fig. 3c and d) the NHEJ dominance is evident, with almost no repair seen. In principle, whilst not fitting with the experimental results, this is the expected result from the model. This dominance occurs since Ku70/80, the initial responder for NHEJ repair, is known to be highly abundant^55^ and has been shown to have fast recruitment kinetics^34,35^. These protein characteristics have been implemented within the *in silico* model, resulting in almost all repair being locked into the NHEJ repair pathway within an NHEJ-deficient cell system, giving rise to a large number of persistent un-repaired ends.

### Scenario C - The ‘continuous competition’ approach

Scenario C has better goodness of fit metrics than Scenario B, but worse than Scenarios A and D (Table 1). In both the WT and Lig4-deficient systems (Fig. 3b and d) it is seen that the simulated data for Scenario C have demonstrated a slower repair rate than the experimental data. Though the amount of repair in the deficient systems is in better agreement with the experimental data than Scenario B, there is still not enough total HR repair within the 8-hour time frame. These slower kinetics are due to re-competition between Ku70/80 and CtIP still being predominantly won by Ku70/80’s faster recruitment kinetics. This results in several attempts of NHEJ before CtIP can attach and resect the end allowing HR to attempt repair. The repair kinetics simulated for the XLF-deficient system is in strong agreement (${\chi }_{red}^{2}=0.40$) with the experiential data set.

### Scenario D - The ‘entwined pathway’ approach

The simulated results for Scenario D are similar, in kinetics and shape, to those from Scenario A with the advantage of not having to be a directed system. Overall, Scenario D has the best goodness-of-fit metrics across all tests (${\bar{\chi }}_{red}^{2}=2.92$, RMSE = 8.06, DTW = 29.01). In the WT-system (Fig. 3b) the scenario closely mimics the shape of the experimental data with improved goodness-of-fit metrics than Scenario A. In the XLF-deficient system (Fig. 2c) the results closely mimic that of Scenario A with slightly slower repair rates. In a similar manner of Scenario A the repair rate post 4 hours is faster than the experimental data which may be due to the HR model missing any form of stalling mechanisms post resection. In the Lig4-deficient system (Fig. 3d) there is again a similar shape to Scenario A with larger deviations at later time points, including noticeably slower kinetics at 8 hours. Compared to Scenario A, there is a higher chance of DNA-PKcs synapsis stabilisation due to the lack of enforcement of HR after a failure of NHEJ and thus increased un-repaired stable complexes without Lig4 to ligate the break.

### Scenario D - Implications of the additional mechanisms

**Figure 4:**
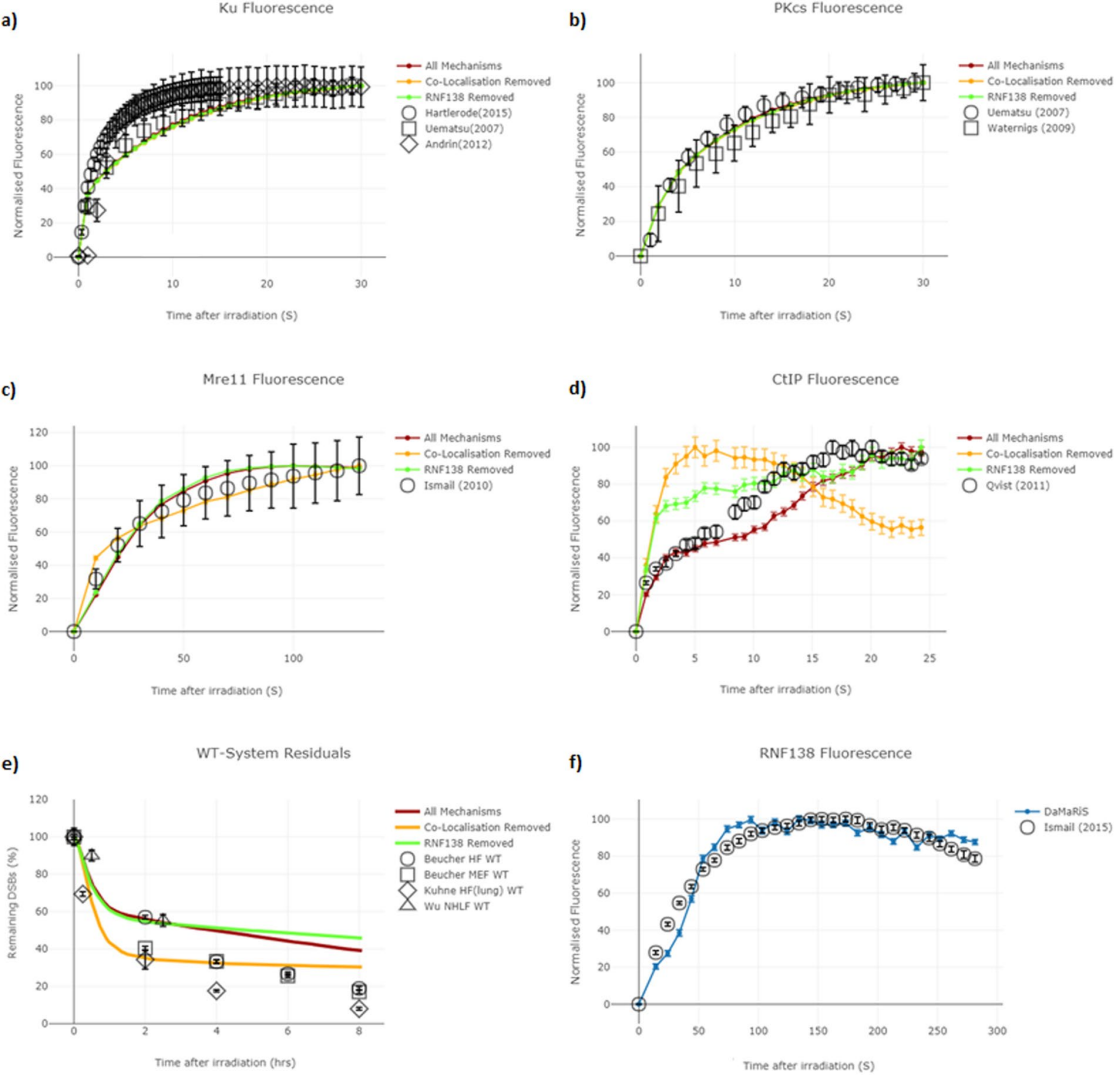
Scenario D mechanism evaluation - the differences in model behaviour are shown for “All Mechanisms” (red line) which includes both MRN co-localisation and RNF138 recruitment, “Co-Localisation Removed” (orange line) which has the role of MRN co-localisation removed and “RNF138 Removed” (green line) which has the role of RNF138 removed. (**a**) Ku immunofluorescence recruitment graph, within the simulation Ku is assumed to persist until synapsis stabilisation or is explicitly removed by RNF138. (**b**) DNA-PKcs immunofluorescence recruitment graph, within the simulation DNA-PKcs is assumed to persist until synapsis stabilisation or is removed along with Ku by RNF138. (**c**) Mre11 (which is representative of the MRN complex) immunofluorescence recruitment graph. (**d**) CtIP immunofluorescence recruitment graph. (**e**) Residual DNA damage graph, experimental data is from *γ*-H2AX foci data and simulation data represents number of breaks which has not yet been repaired. f) RNF138 immunofluorescence recruitment graph. All error bars are the ±SEM for both simulated and experimental data. In Sub-Figure (**f**) the ±SEM is represented as the width of the line. The error displayed is representative of 250 repeated simulations for protein recruitment data and 50 repeated simulations for un-repaired data, each with their own independent exposures on different cells.

The competition, in Scenario C, between Ku70/80 and CtIP recruitment kinetics does not allow for HR repair at the same rate seen in the experimental data. It is known that the Mre11-Rad50-Nbs1 (MRN) complex is also required for CtIP activation and resection^41,56^. However, explicit modelling of the recruitment kinetics of MRN was not established within Scenario A, B, or C and instead explicit modelling of CtIP was included with the assumption that MRN would be present at this point. Furthermore, the previous scenarios were unable to match with experimental results of Mre11 recruitment. However, upon enabling the recruitment of MRN alongside attempting the NHEJ pathway, the simulation recruitment kinetics have good agreement with the experimental recruitment kinetics (Fig. 4c). This addition of MRN co-localisation with NHEJ proteins results in a naturally occurring bias, that if MRN has attached during the NHEJ repair attempt, then the CtIP attachment is more likely to attach at points of re-competition.

**Figure 5:**
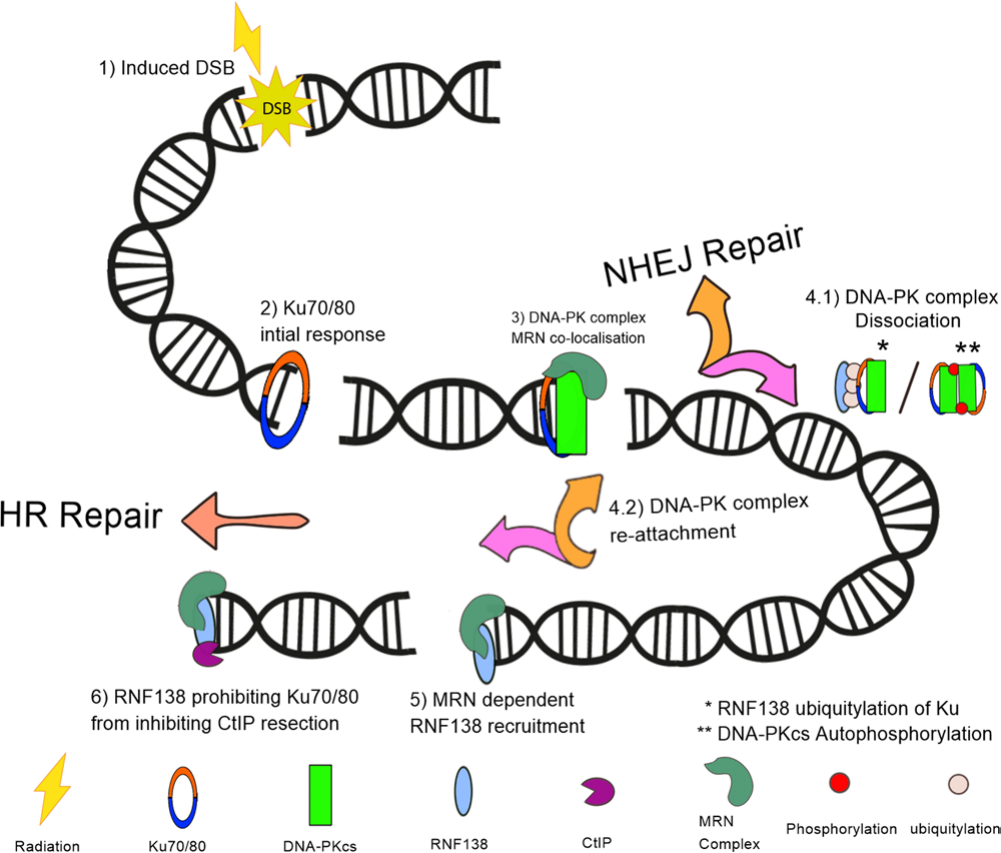
Schematic of Scenario D– The ‘entwined pathway’ approach of repair choice. Positions 1–6 represent steps along the DNA DSB repair pathway. (1) Radiation induces DSB damage. (2) Due to its high abundance and affinity for DNA ends, on average Ku provides the first response to the DSB. (3) DNA-PKcs can attach in a Ku-dependent manner and/or MRN can co-localise with Ku/DNA-PKcs. (4.1) Either the DNA-PK synapsis can be stabilised allowing for NHEJ repair to follow or the DNA-PK complex can dissociate through either autophosphorylation or RNF138 ubiquitylation. (4.2) Either NHEJ can be attempted again by the recruitment of Ku and then DNA-PKcs, or steps can be taken towards resected-mediated repair. (5) RNF138 is recruited in an MRN dependent manner. (6) RNF138 prohibits Ku attachment providing enough time for CtIP resection to take place and HR repair to follow. As the repair model is Monte Carlo-based, with various progression points at each step, the above is one possible repair route available in the entwined pathway model. However, this route has been demonstrated to be important for matching protein recruitment kinetics (Fig. 4) and highlights an NHEJ and HR symbiotic relationship.

The incorporation of RNF138-dependent removal of Ku70/80 and the DNA-PK complex within Scenario D has subtle effects when compared to those of co-localisation (Fig. 4e). Dissociation of Ku70/80 and the DNA-PK complex which shields the DNA-end from CtIP-based resection increases the likelihood of HR repair taking place. Furthermore, as RNF138 is recruited in an MRN-dependent manner, the effects are increased with the MRN co-localisation mechanism applied. In Scenario D, the RNF138 protein is recruited at a time constant approximately 80 times larger than that of Ku and DNA-PKcs recruitment and 3 times larger than MRN recruitment. This larger time constant results in RNF138 increasing at later time points of the simulation. This translates to an increased bias towards HR at later time points in the simulation. There is a complex behaviour between the mechanisms and CtIP recruitment (Fig. 4d): without co-localisation, there is not the required increasing relationship with time required to match the experimental data, whereas if RNF138 is removed the increasing relationship with time is present, but the initial recruitment is too fast. The best fit to the CtIP recruitment data is achieved through the inclusion of both co-localisation and RNF138 (Fig. 5).

## Discussion

The analysis of each scenario in turn, when compared to experimental literature results, has highlighted the poor fit of competitive only pathways (Scenarios B and C) to model the repair choice process (Table 1). However, we show that with the inclusion of co-localisation of the MRN complex, a complex typically thought of as being involved in HR, with initial NHEJ proteins (Scenario D), it is possible to better mimic the repair kinetics of the Beucher *et al*., experimental data. Scenario D is not exclusively competitive and utilises co-localisation of proteins to allow for explicit modelling of MRN recruitment. Although Scenario D is the most favoured repair choice, it should be stated that Scenario A, the ‘NHEJ first’ approach, also fits the experimental results well. However, Scenario A is heavily directed as we force the model to use NHEJ and then utilise HR only if the repair fails. Furthermore, while Scenario A can mimic repair rates similar to Scenario D, without co-localisation the explicit modelling of MRN recruitment is not possible and CtIP protein recruitment is delayed in the simulation which deviates from the experimental recruitment data (see Supplementary Data – Fig. S1). The inability to fit the MRN and CtIP recruitment kinetics could be easily overlooked in less mechanistic approaches where protein level progression is being omitted. In contrast, Scenario D retains the known faster recruitment kinetics of Ku whilst not having to apply system constraints that cannot be substantiated by literature-reported mechanisms.

The introduction of additional mechanisms to Scenario D required careful consideration of all the implications on the model. RNF138-dependent removal of Ku and DNA-PKcs at DNA ends with MRN attached was fitted to the immunofluorescence recruitment of RNF138 (Fig. 4f). Whilst the removal of Ku70/80 from the DNA end was more clearly set-out within the literature^40^, the mechanism of removal of DNA-PKcs along with Ku70/80 back to a naked DNA end was established from an amalgamation of literature results. *Firstly*, it is widely understood that DNA-PKcs is recruited in a Ku-dependent manner^35,57^. *Secondly*, it is believed that Ku70/80, under regular cellular conditions^58^, helps retain DNA-PKcs at the end of the DSB^59^*. Finally*, Ismail *et al*., 2015^40^ demonstrated that the recruitment of RNF138 is not dependent on Ku70/80 foci formation, resulting in our interpretation that if Ku70/80 was somehow protected by DNA-PKcs from RNF138-dependent removal, then it would be adversely affected by the DNA-PKcs recruitment that arises from the Ku70/80 foci formation. The establishment of MRN complex co-localisation was also an amalgamation of literature findings. *Firstly* Britton *et al*., 2013^39^ showed co-localisation through simultaneous visualisation of Ku and NBS1 (part of the MRN complex) foci, and *secondly* Zhou *et al*., 2017^36^ found the presence of Ku/DNA-PKcs does not impact the recruitment of MRN/ATM, a premise further supported by earlier work from Hartlerode *et al*., 2015^34^ who showed MRN and Ku recruitment to be independent of one another. The observed agreement in Mre11 recruitment kinetics post-adjustment of the model for co-localisation gives further confidence in the added mechanism (Fig. 4). The addition of these mechanisms has only been applied for Scenario C’s ‘continuous competition’ approach to form Scenario D. However, these additions were evaluated for both Scenario A and B to evaluate if further simulations were required. In Scenario A’s ‘NHEJ first’ approach, the co-localisation would be non-consequential due to the directed nature of enforcing NHEJ before HR. Also, the additional dissociation points would lead to a minimal change in the WT and Lig4-deficient systems with slightly quicker repair rates. Therefore, it is likely to result in noticeable differences in XLF-deficient cells which would quicken the repair rate further beyond the already to fast kinetics observed (Fig. 3c), negatively impacting the fit. In Scenario B’s ‘no way back’ approach, both the co-localisation and additional dissociation points would be non-consequential as there is no cross-talk between post initial protein loading. For these reasons, the additional mechanisms incorporated were limited to Scenario C’s ‘continuous competition’ approach to form Scenario D with the observed effects shown in Fig. 3.

This work has demonstrated how subtle changes in the repair choice can lead to substantial changes in the repair kinetics and hence its importance in repair modelling. Furthermore, the study has demonstrated that new mechanisms proposed in the literature can be applied and evaluated in a timely manner using the DaMaRiS framework. The difficulty in modelling using a mechanistic approach is that as each step is modelled independently, missing or incorrectly modelled steps can lead to the system deviating from experimental results. As no single group have published work for every required mechanism included in this *in silico* model, the experimental data used to model each step comes from various sources and thus inter-experimental uncertainty will be present. Furthermore, the final data set used to examine our *in silico* model is again from a different source to those used to build the model, which whilst being beneficial in avoiding systematic error is hindered by further inter-experimental uncertainties. The advantage of a step-by-step mechanistic model is that each step can be independently validated against experimental data.

Though some causes of the differences between the simulated and experimental data are explained above, others can be explained with the examination of the model response to the different scenarios and cell systems tested. Within the XLF-deficient system, the progression to synapsis stabilisation is removed^46,54^; this means that DNA-PKcs synapsis dissociation will occur given enough simulation time. Upon dissociation of DNA-PKcs synapsis, HR is enforced (within Scenario A) or is allowed to re-compete (within Scenario C & D), and repair, whilst being longer, will be achieved given enough time. This resultant repair produces the observed linearity of the simulation data and the overall deviation from the experimental data beyond 2 hours. To be able to emulate the results seen in the XLF-deficient system either further mechanisms for un-repaired DNA ends to form should be present within HR, or XLF-deficiency is not an absolute determinant of synapsis stabilisation, but does perturb the ability to ligate the break. Whilst XLF is known to be important within NHEJ repair, its purpose is not as clearly defined^60^ as that of Lig4; this may be why the simulation reproduces better the Lig4-deficient system than the XLF-deficient system. Therefore, the choice to attempt modelling XLF-deficiency in this study required extra consideration. *Firstly*, it has been shown that XLF availability impacts NHEJ and not HR^61^, so the alterations should only be made to the NHEJ pathway. *Secondly*, XLF is not merely a tool to quicken NHEJ, as when depleted it can significantly impact the repair capabilities of the system^31,60^. *Thirdly*, XLF is thought to provide additional stability to Lig4 and XRCC4 at the final steps of NHEJ repair^61^, is likely to be present prior to Lig4^60^ and may even be present early enough to help achieve synapsis formation^54,62^. Given the ambiguity regarding the explicit use of XLF in NHEJ repair, additional simulations of XLF-deficient systems are presented in the Supplementary Data (Fig. S7). The study included the addition of experimental literature data from two WT and one Lig4 deficient cell lines for analysis of the variation one may expect from different laboratory set-ups (Fig. 3). The WT cell system data set from Kuhne *et al*. and Wu *et al*. are examples of both faster and slower repair kinetics respectively. Interestingly the Lig4 syndrome cell system from the Kuhne *et al*. data set shows substantially faster kinetics than those observed through simulation. This is most likely due to the cell line not being entirely devoid of the Lig4 protein which would allow for a small amount of NHEJ repair^50^. In order to evaluate this data set accurately, partial progression inhibition would be needed within the model.

The analysis of repair choice between each scenario is predominately unaffected by the modelling post resection within the HR repair pathway. However, the simplification of modelling the progression of DNA ends that have been resected through to being repaired using a single time constant, *τ*_*RR*_, may be responsible for some of the discontinuities when compared with experimental literature data. It is expected that a more detailed model of HR repair post-resection, which includes the repair rate implications of Rad51-filament seeking out the sister chromatid, may help to better mimic the shape of the repair kinetics seen in experimental results whilst being able to maintain the mechanistic approach. However, modelling HR repair in such fine detail to uphold the current step-by-step mechanistic modelling approach is a large project and to our knowledge, no such model currently exists. Furthermore, several of the steps in HR are lacking detailed descriptions, such as filament seeking^63^, misrepair^64^ and sub-pathway choice^44^, increasing the challenge of modelling and further encouraging studies such as this one to improve our mechanistic understanding of DNA repair and therefore how best to model it.

The requirement for all DSBs to be repaired via either NHEJ or HR is a simplification. In practice there are several other, less commonly utilised, repair pathways able to address DSBs. Alternatively, DSB repair could be classified as either being resection-dependent or resection-independent: within the scope of the presented work this would be analogous to HR and NHEJ respectively. As the current model does not include Alternative NHEJ (Alt-NHEJ) or Single Strand Annealing (SSA), both of which are resection-dependent DSB repair pathways, any presence within the experimental data would be classified by the model as HR. Whilst Alt-NHEJ and SSA are believed to be less utilised, there is an increase of use for deficient cell lines, specifically NHEJ deficient cell lines^65,66^, which are present in this study. Furthermore, before any analysis of the fidelity of repair from the model can be made, the biological impact of these additional resection-dependent DSB repair mechanisms should be considered. Whilst HR, Alt-NHEJ, and SSA are commonly categorised as resection-dependent repair, their processes are very different. It is thought that HR is a relatively error-free process, while both Alt-NHEJ and SSA are considered to be extremely error-prone, often resulting in large deletions^67^.

Though the mechanisms investigated in this study encompass how the repair choice should be interfaced within an *in silico* model, there are additional factors which are thought to influence repair choice which have not been included. The additional factors are either tied to the DNA geometry or the detail of the damage that is trying to be repaired. The currently simulated model takes place on an entirely homogeneous spread, whereas it is known that the cell nucleus has a heterogeneous spread of DNA geometry formed by euchromatin and heterochromatin regions. It is believed that these regions have biases towards particular repair pathways^42^, an aspect omitted from the current study. Furthermore, the DNA density will also have an impact on the proximity between DSBs, which has been previously scored through the ‘ Cluster Density’^7^, a known aspect of repair fidelity, but one which will also impact the quantities of repair as further proximity-based mechanisms are incorporated within the model (e.g. sister chromatid seeking). Additional to the DNA geometry, a known factor in the production of DNA damage density is the incident radiation source, dose and the linear energy transfer (LET). This study utilises a range of experimental set-ups using various radiation sources, but the simulations are all based on 1.77 keV/*μ*m LET mono-energetic proton irradiation of 2 Gy. There is an established belief within the literature that repair choice is influenced by the damage complexity^43^, although the mechanisms which produce this effect are still to be established. At present, there is nothing within our model that would lead us to observe this sensitivity, though the literature is being carefully observed for new mechanisms to be proposed which can be incorporated into our *in silico* models and further exploration of repair choice can be carried out.

## Conclusion

This study has demonstrated the complexities of modelling repair choice within a mechanistic *in silico* model. The ability to match the recruitment kinetics of key proteins whilst piecing together the possible progression and dissociation of said proteins, based on what has been seen experimentally, is challenging. Overall, this study has demonstrated that the ‘entwined pathway’ approach (Fig. 5), for modelling the competition between the two main pathways of DSB repair, results in the most effective fit to the evaluated published data. Furthermore, it has been shown in this study that co-localisation of MRN with initial NHEJ proteins and RNF138-dependent removal of Ku are required in order to achieve a model which can emulate both the protein recruitment and repair kinetics from experiments. Through the explicit modelling of protein recruitment the DaMaRiS model is able to meet more constraints whilst remaining mechanistic. This approach allows the combination of many experimentally proposed mechanisms into a single system to explore and evaluate how they interact with one another.

## Supplementary information

SUPPLEMENTART

## Data Availability

The data sets generated and/or analysed during the current study are available from the corresponding author on reasonable request.
