# Supplementary material for: Mechanistic modelling supports entwined rather than exclusively competitive DNA double-strand break repair pathway
Source: Sci Rep. 2019 Apr 23;9:6359. doi: 10.1038/s41598-019-42901-8 (PMC6478946; doi:10.1038/s41598-019-42901-8)
Supplement: Supplementary file 1 — SUPPLEMENTART [file 41598_2019_42901_MOESM1_ESM.pdf]

The repair pathway choice of DNA double-strand breaks: insights from mechanistic modelling

S. P. Ingram [1,2], J. W. Warmenhoven [1,3], N. T. Henthorn [1,3], E. A. K. Smith [1,2], A. L. Chadwick [1], N. G. Burnet [1,3], R. I. Mackay [2,1], N. F. Kirkby [1,3], K. J. Kirkby [1,3] & M. J. Merchant [1,3]

[1] Division of Cancer Sciences, Faculty of Biology, Medicine and Health, The University of Manchester, UK
[2] Christie Medical Physics and Engineering, The Christie NHS Foundation Trust, Manchester, UK
[3] The Christie NHS Foundation Trust, Manchester Academic Health Science Centre, Manchester, UK

Supplementary Information

Protein Recruitment Kinetics. Accurate protein recruitment kinetics are essential for the total repair timings. This section details the results of fitting recruitment time constants in the *in-silico* model and how they compare to the recruitment kinetics seen in literature from fluorescence foci imaging. It should be noted that no absolute quantification efforts have been made. Instead, the relative build-up of foci through to levels of a plateau as a function of time have been used. There is a comparison of the changes in simulated recruitment kinetics throughout the tested repair choice scenarios for Ku, DNA-PKcs and CtIP.

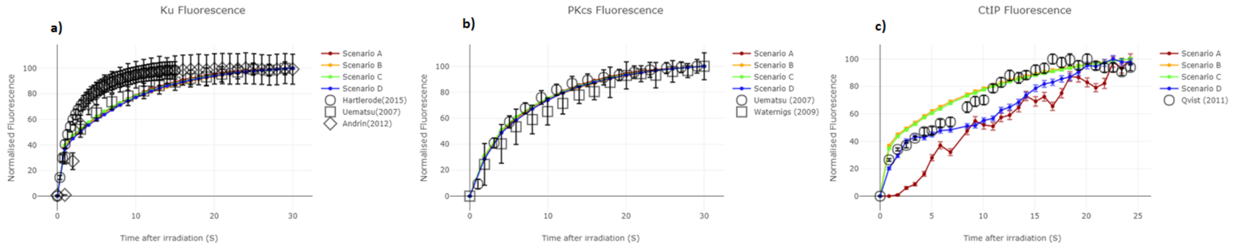

Figure S1: protein recruitment kinetics scenario comparison - a) shown insensitivity of the recruitment kinetics of Ku70/80 for the tested scenarios. b) shown insensitivity of the recruitment kinetics of DNA-PKcs for the tested scenarios. c) shown variation in the recruitment kinetics of CtIP for the tested scenarios. All error bars are ±SEM from either the reported experimental data or from 200 repeated simulations each with their own independent exposures on different cells.

Flexibility of scenarios (Variations in τ_{RR}). Throughout the repair choice scenario testing the time constant for the progression between DNA end that has been resected and DNA end repaired (τ_{RR}) has been allowed to vary. This allowed variable leads to a spectrum of possible repair kinetics. The allowed variation for each cell system tested is shown in Figure S2, Figure S3, Figure S4 and Figure S5 for Scenarios A, B, C and D respectively. The possible variation is shown by varying τ_{RR} for multiple simulations between 100s and 100,000s.

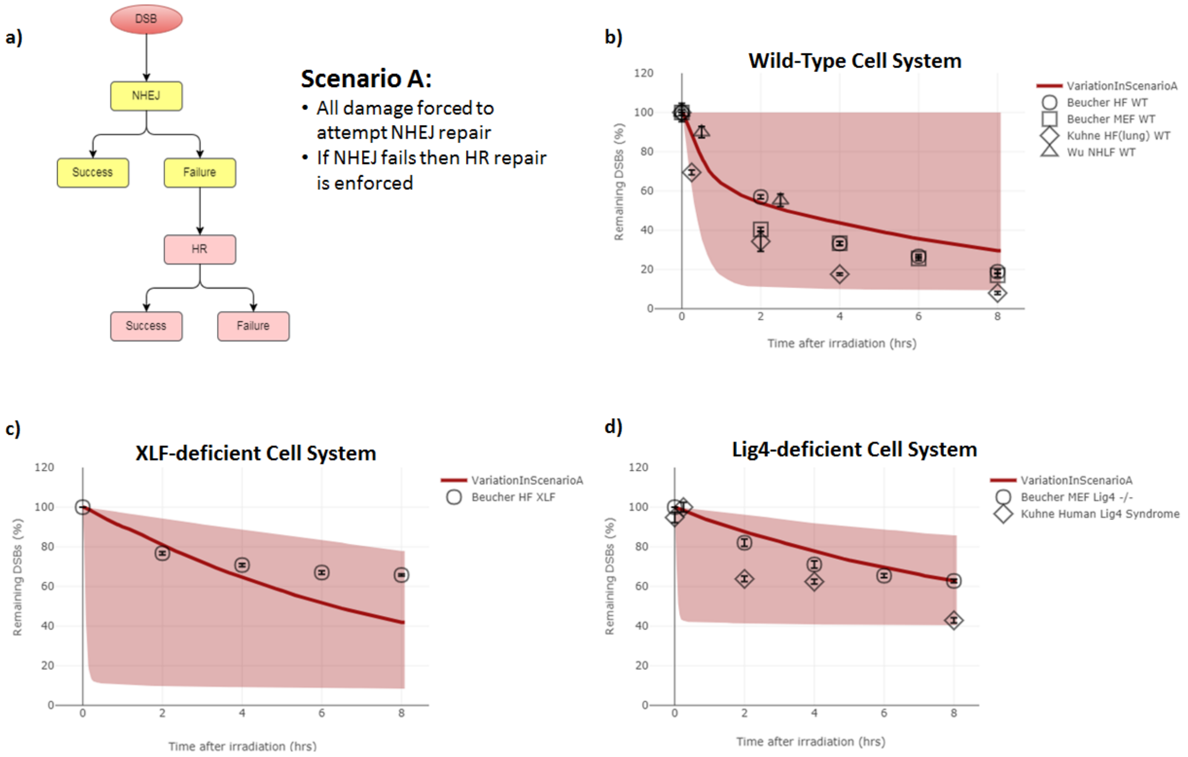

Figure S2: Flexibility in Scenario A - the simulated repair kinetics displayed in Figure 3 is shown as the emboldened red line. The coverage of red colour represents the possible repair kinetics when varying τ_{RR} for multiple simulations between 100s and 100,000s. b), c) and d) represent the Wild-Type, XLF-Deficient and Lig4-Deficient cell systems respectively, with a) being a simplified schematic of the tested repair choice scenario. The available variation of Scenario A results shows that the majority of data-points can be mimicked though this would require multiple τ_{RR} time points to be used. Whilst the use of multiple τ_{RR} time points would not be used, the understanding that you can achieve the flexibility to mimic experimental values gives merit to Scenario A's repair choice representation.

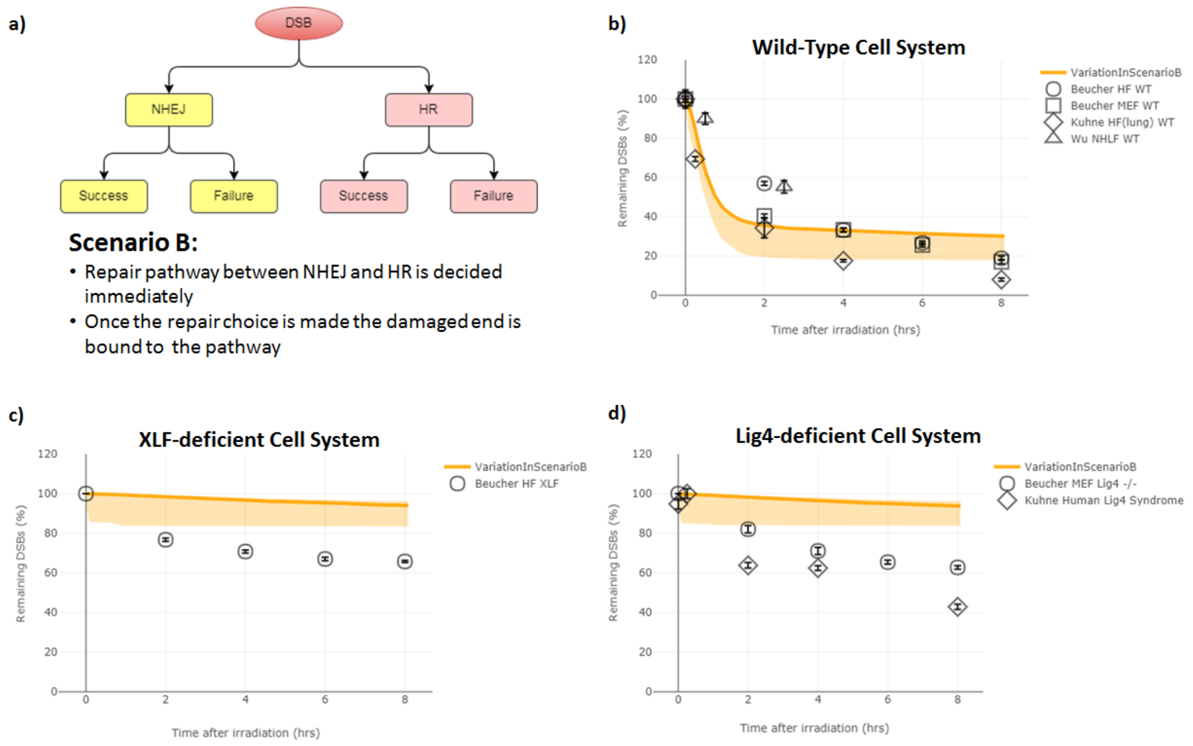

Figure S3: Flexibility in Scenario B - the simulated repair kinetics displayed in Figure 3 is shown as the emboldened orange line. The coverage of orange colour represents the possible repair kinetics when varying τ_{RR} for multiple simulations between 100s and 100,000s. b), c) and d) represent the Wild-Type, XLF-Deficient and Lig4-Deficient cell systems respectively, with a) being a simplified schematic of the tested repair choice scenario. The shown variation in all cell systems demonstrates that Scenario B is unable to mimic both the values and shape of the experimental data. This supports the argument of a purely competitive system with no repair pathway cross-talk being unrepresentative of repair choice.

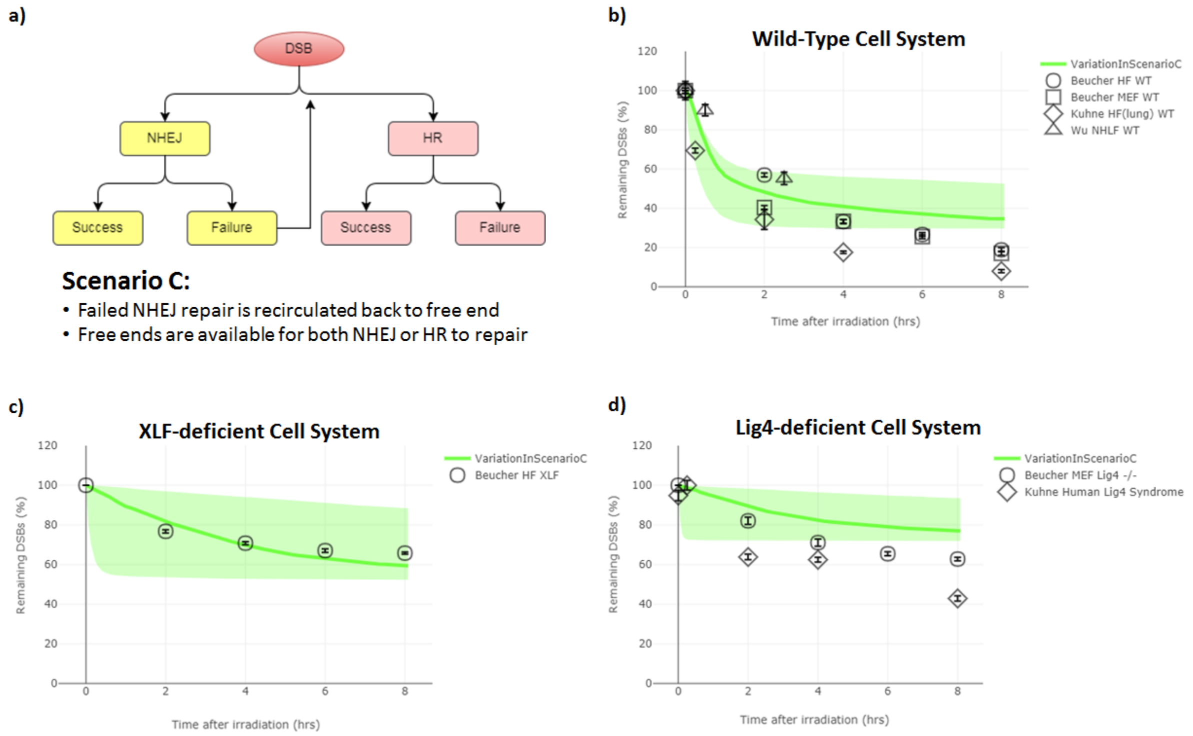

Figure S4: Flexibility in Scenario C - the simulated repair kinetics displayed in Figure 3 is shown as the emboldened green line. The coverage of green colour represents the possible repair kinetics when varying τ_{RR} for multiple simulations between 100s and 100,000s. b), c) and d) represent the Wild-Type, XLF-Deficient and Lig4-Deficient cell systems respectively, with a) being a simplified schematic of the tested repair choice scenario. Whilst the repair kinetics of the XLF-deficient cell system c) can be well represented by Scenario C, the possible variation achievable in the WT a) and Lig4-deficient d) cell systems show that repair kinetics at 6 and 8 hours cannot be mimicked. This failure supports the argument that a purely competitive system with cross-talk through re-competition is not representative of repair choice.

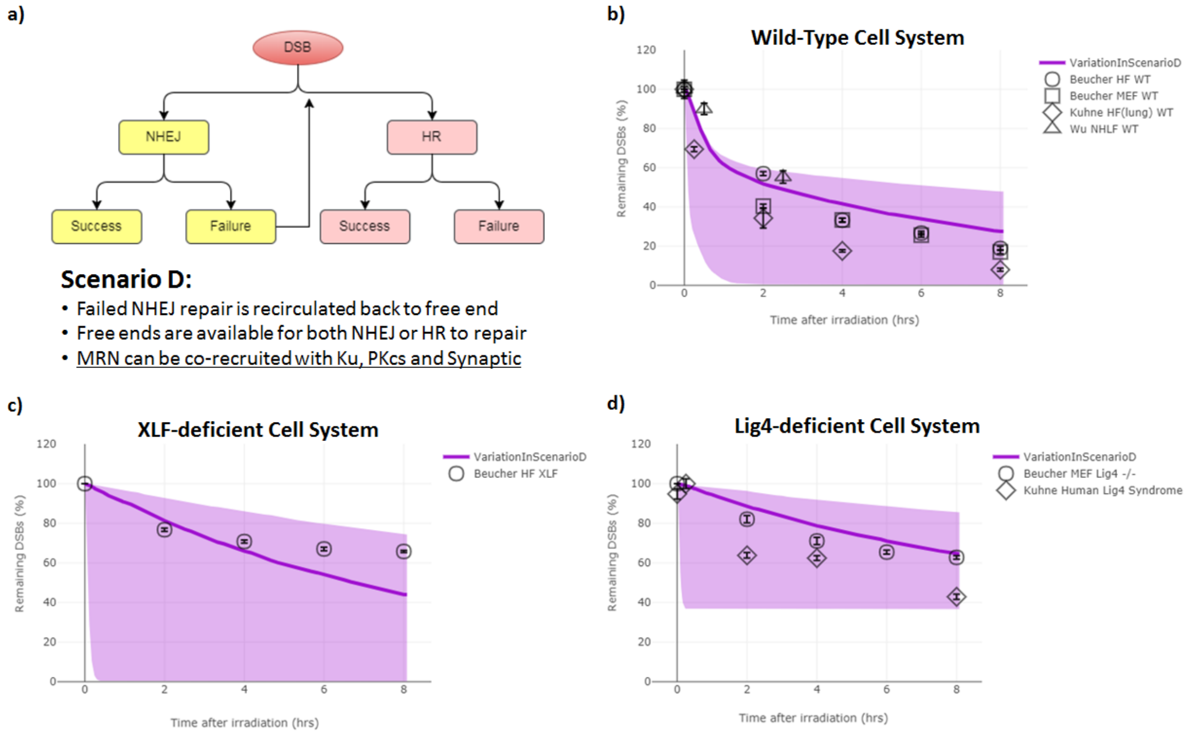

Figure S5: Flexibility in Scenario D - the simulated repair kinetics displayed in Figure 3 is shown as the emboldened purple line. The coverage of purple colour represents the possible repair kinetics when varying τ_{RR} for multiple simulations between 100s and 100,000s. b), c) and d) represent the Wild-Type, XLF-Deficient and Lig4-Deficient cell systems respectively, with a) being a simplified schematic of the tested repair choice scenario. Similarly to Scenario A the achievable variation shown in Scenario D shows it can mimic the majority of experimental data points. This flexibility gives merit to the scenario and a system which utilises non-directed competition along with co-localisation of repair proteins to represent the repair choice.

Variations of XLF-deficiency modelling. This study models XLF-deficiency as the failure of synapsis stabilisation between two DNA-PK complexes. An alternative model of XLF-deficiency was explored where the deficiency resulted in the inability to ligate DNA ends, similar to the modelled Lig4-deficient system. This alternative approach was evaluated for a range of synapsis stabilisation impairments, where the time constant applied to synapsis stabilisation was increased (Figure S6). The simulated results for the XLF deficiency leading to the inability to ligate with varying levels of synapsis stabilisation impairment is shown in Figure S7.

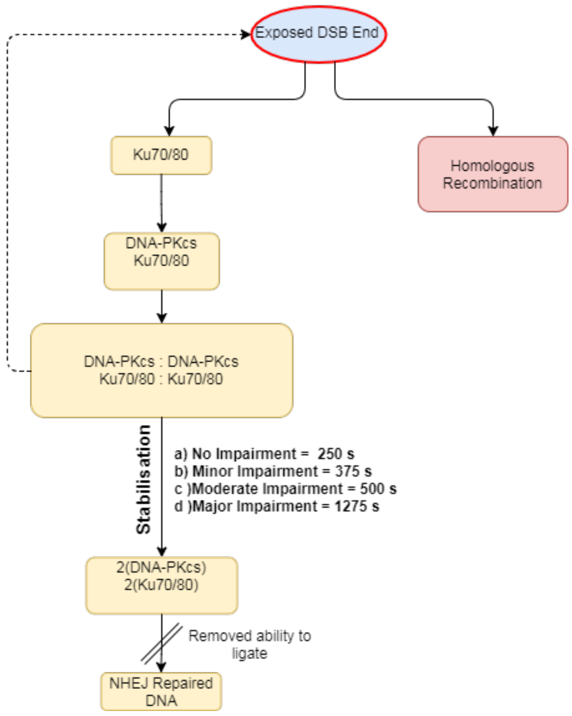

Figure S6: Schematic of variations in modelling XLF-deficiency - diagram depicting the alternative approach to modelling XLF-deficiency where ligation is made impossible and the stabilisation of the synapsis complex is impaired taking longer to complete. The level of impairment ranges from no impairment (250 s), minor impairment (375 s), moderate impairment (500 s) and major impairment (1275 s). The progression to "Homologous Recombination" varies in the same manner as to what is shown in Figure 2 for each repair choice scenario.

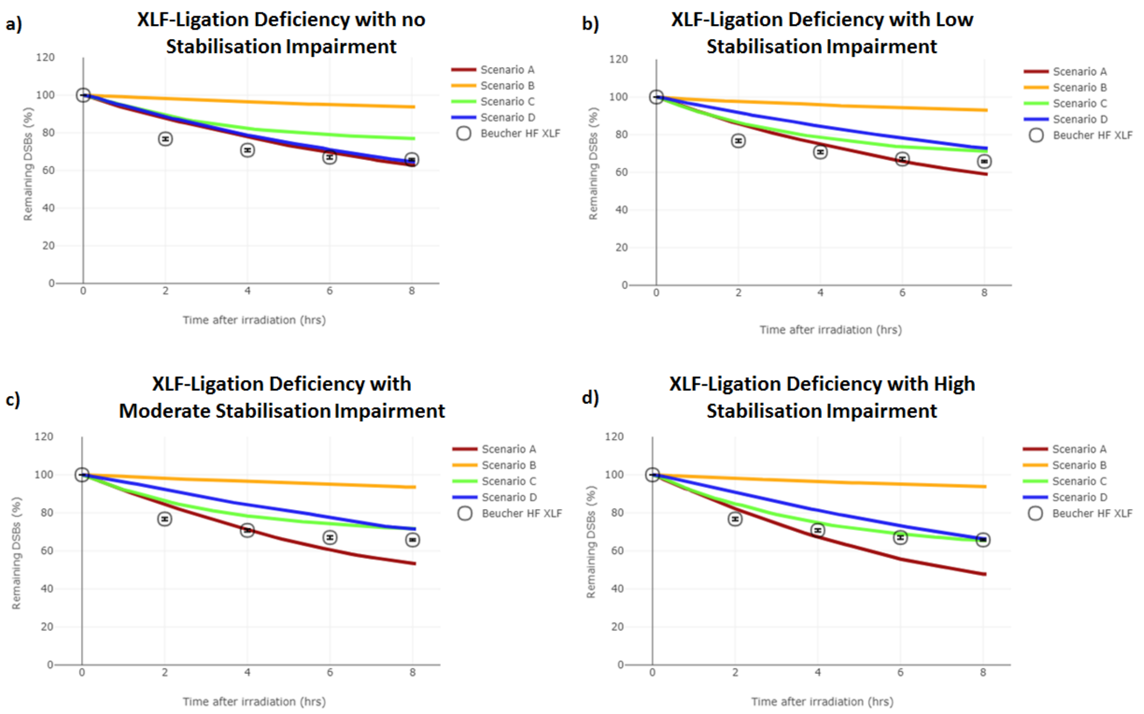

Figure S7: Simulated results of variations in modelling XLF deficiency - results are shown for each scenario with the same optimised time constants used in Figure 3 with the variation shown in Figure S6. The exact time constants used for each repair choice scenario is shown in Figure S8-S11. a) no stabilisation impairment present, results are the same as the simulated Lig4-deficient system in Figure 3, but have been plotted against experimental XLF-deficient data. b) minor stabilisation impairment, increasing the the stabilisation time constant by 1.5 times. c) moderate stabilisation impairment, increasing the stabilisation time constant by 2.0 times. d) major stabilisation impairment, increasing the stabilisation time constant by 5.0 times. The error in the simulated data is the ±SEM is displayed as the line width for 50 repeated simulations each with their own independent exposures on different cells.

Detailed diagrams of scenarios. The repair choice scenarios modelled have been optimised to fit with protein recruitment kinetics where possible. The diagrams below are schematic depictions of the simulated repair pathways including the time constants used. For the different cell systems modelled (Wild-Type, XLF-deficient and Lig4-deficient) the time constants remain fixed for each scenario with only set progression points removed, as shown in Figure 2. Protein names listed within the boxes represents the stages at which it is believed the protein is still present and therefore would be counted in the simulated recruitment kinetics graphs (Figure S1). Boxes in grey represents stages of the model which were not explicitly modelled. With dotted lines representing the time constant which encompasses non-explicitly modelled steps. Yellow boxes represent Non-Homologous End Joining steps, red boxes represent Homologous Recombination steps and blue boxes represent DNA end processing steps. The "Exposed DSB End" with the red lining represents the starting point of the repair simulation as all DNA ends are placed here at the start.

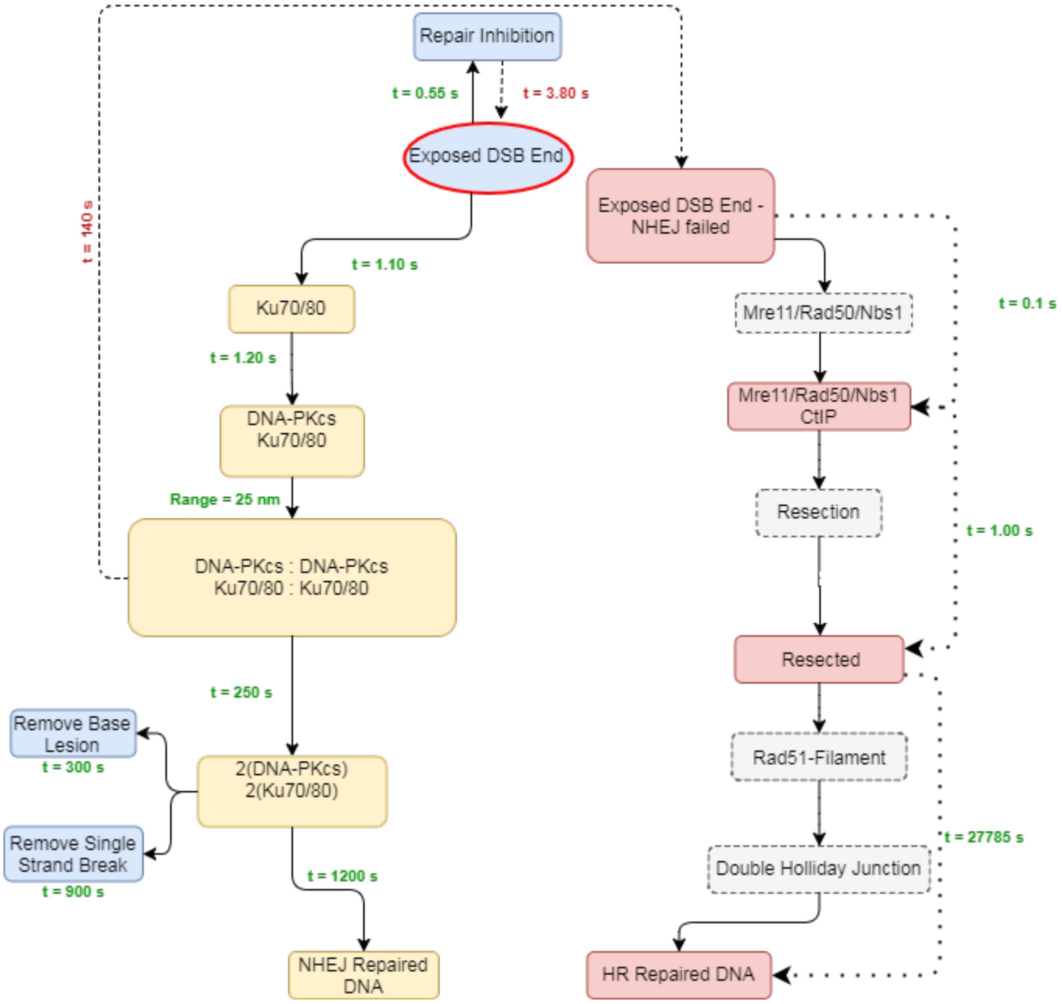

Figure S8: Detailed schematic of Scenario A - diagram shows the time constants used for modelling Scenario A with the DaMaRiS framework (v0.3). Progression time constants are in green text and regression time constants are in red (with a dashed line).

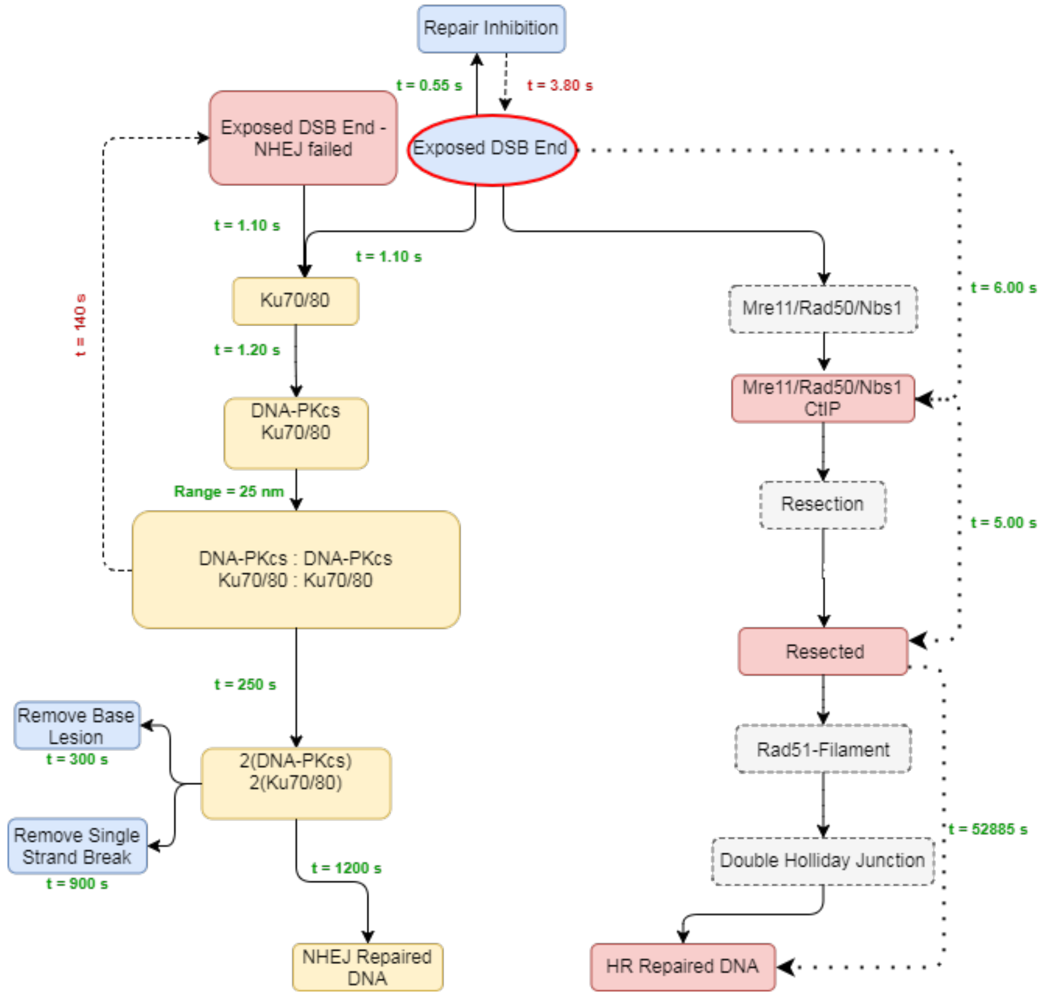

Figure S9: Detailed schematic of Scenario B - diagram shows the time constants used for modelling Scenario B with the DaMaRiS framework (v0.3). Progression time constants are in green text and regression time constants are in red (with a dashed line).

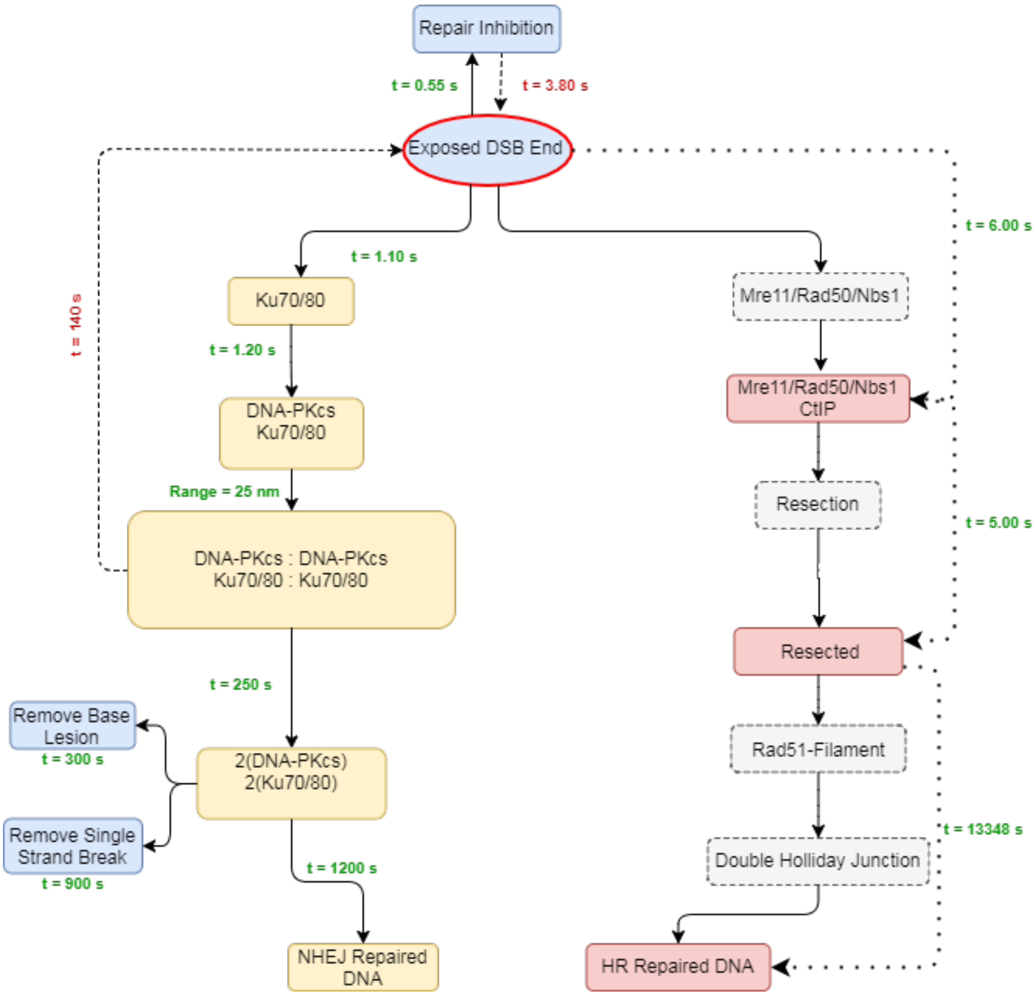

Figure S10: Detailed schematic of Scenario C - diagram shows the time constants used for modelling Scenario C with the DaMaRiS framework (v0.3). Progression time constants are in green text and regression time constants are in red (with a dashed line).

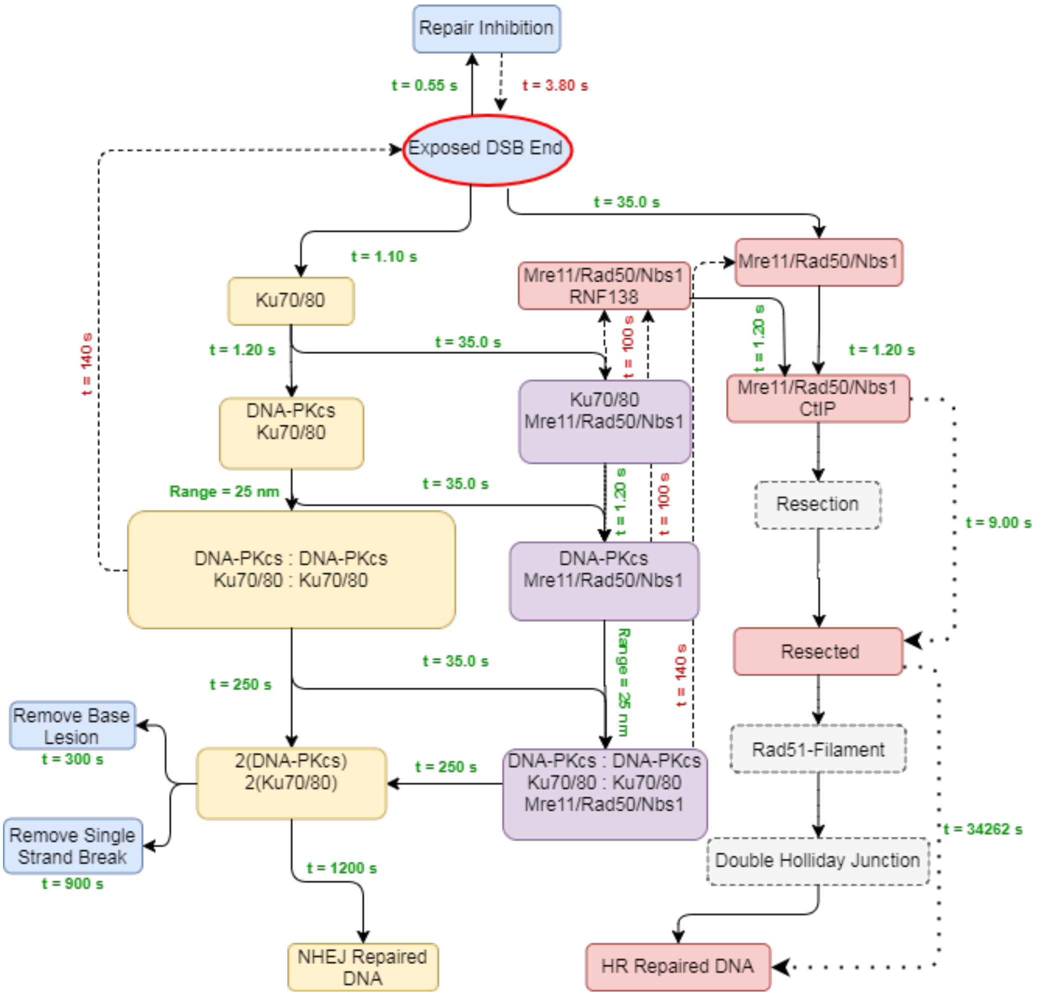

Figure S11: Detailed schematic of Scenario D - diagram shows the time constants used for modelling Scenario D with the DaMaRiS framework (v0.3). Progression time constants are in green text and regression time constants are in red (with a dashed line).
